# Impact of the COVID-19 Pandemic on Splanchnic Thrombosis Mortality: A United States National Analysis (2018-2023)

**DOI:** 10.7759/cureus.104562

**Published:** 2026-03-02

**Authors:** Mohammad Alali, Ahmed Kunwer Naveed

**Affiliations:** 1 Internal Medicine, Henry Ford Macomb Hospital, Macomb, USA

**Keywords:** covid-19, epidemiology, mesenteric ischemia, mortality trends, portal vein thrombosis, splanchnic thrombosis

## Abstract

Background

Coronavirus disease 2019 (COVID-19) has been associated with systemic effects that may influence thrombotic risk. While venous thromboembolism has been widely reported, national mortality trends for splanchnic thrombosis, including portal vein thrombosis, Budd-Chiari syndrome, mesenteric venous thrombosis, and mesenteric ischemia, remain poorly characterized.

Methods

The CDC WONDER Multiple Cause of Death database was queried for 2018-2023. Annual deaths, population counts, and crude mortality rates per 100,000 persons were extracted and stratified by year and sex. Mortality patterns during the pre-pandemic period (2018-2019) were compared with the pandemic/post-pandemic period (2020-2023) using descriptive trend analysis.

Results

A total of 32,738 deaths were attributed to splanchnic thrombosis during the study period. Annual deaths increased from 4,819 in 2018 to 5,986 in 2023. The pre-COVID-19 annual average was 4,931 deaths compared with 5,719 deaths during 2020-2023, representing a 16.0% relative increase. Crude mortality rates rose from 1.5 per 100,000 (2018-2019) to 1.7-1.8 per 100,000 (2021-2023). Increases were observed in both sexes.

Conclusions

Mortality from splanchnic thrombosis increased during the COVID-19 era. These findings demonstrate a temporal rise in mortality; however, the study design is descriptive and does not establish causality. Further research is needed to evaluate potential contributing factors and clinical implications.

## Introduction

Coronavirus disease 2019 (COVID-19) has been associated with systemic inflammation, endothelial dysfunction, platelet activation, and a hypercoagulable state [1]. These pathophysiologic processes have been linked to an increased risk of thrombotic complications, including venous thromboembolism, arterial thrombosis, and microvascular injury [2]. While pulmonary embolism and deep vein thrombosis have been extensively studied during the pandemic, less attention has been directed toward thrombotic events involving the splanchnic circulation.

Splanchnic thrombosis encompasses a spectrum of rare but clinically severe conditions affecting the portal, hepatic, mesenteric, and splenic vasculature. These disorders often present with nonspecific symptoms, which may delay diagnosis and contribute to significant morbidity and mortality. Acute mesenteric ischemia, for example, is associated with mortality rates exceeding 50% when diagnosis is delayed [3]. Similarly, portal vein thrombosis and Budd-Chiari syndrome can result in portal hypertension, hepatic failure, or intestinal infarction [4].

During the COVID-19 era, reports have described thrombotic complications occurring in atypical vascular territories, including the splanchnic circulation [2]. However, the existing literature is largely limited to case reports and small case series. Population-level data evaluating national mortality patterns involving splanchnic thrombosis before and after the onset of the COVID-19 pandemic remain limited.

This study aimed to compare national mortality trends among deaths involving splanchnic thrombosis in the United States between the pre-pandemic period (2018-2019) and the pandemic/post-pandemic period (2020-2023).

## Materials and methods

Data source

This retrospective population-based study utilized mortality data from the Centers for Disease Control and Prevention Wide-ranging Online Data for Epidemiologic Research (CDC WONDER) Multiple Cause of Death database [5]. This publicly available dataset contains US death certificate information, including demographic characteristics, underlying and contributing causes of death, and census-derived population estimates. A known limitation of death certificate data is the potential for misclassification or underreporting of specific conditions; however, such misclassification is unlikely to have changed systematically during the study period.

Study period and population

Deaths occurring between January 1, 2018, and December 31, 2023, were included. All deaths recorded within the United States during this period were eligible for analysis.

Case identification

Splanchnic thrombosis-related deaths were identified using International Classification of Diseases, Tenth Revision (ICD-10) codes listed as either underlying or contributing causes of death [6]. Included codes were I81 (portal vein thrombosis), I82.0 (Budd-Chiari syndrome), I82.2-I82.3 (abdominal venous thrombosis), and K55.0-K55.1 (mesenteric ischemia). These codes were selected a priori based on established diagnostic classifications for abdominal vascular thrombosis and ischemia.

Outcome measures and demographic variables

The primary outcome was annual mortality among deaths involving splanchnic thrombosis. Data were stratified by calendar year and sex. Sex was the only demographic variable included in the analysis because it was consistently available with complete reporting across the study period and allowed for stable subgroup comparisons. Other demographic variables available in CDC WONDER (e.g., age, race/ethnicity, and geographic region) were not included to maintain a focused descriptive analysis and avoid small cell counts or unstable estimates for these relatively rare conditions. Extracted variables included total deaths, population counts, and crude mortality rates per 100,000 persons.

Statistical analysis

Descriptive statistics were used to summarize mortality trends. The pre-pandemic baseline period was defined as 2018-2019 and compared with the pandemic/post-pandemic period of 2020-2023. Average annual deaths were calculated for each period. Relative percent change between periods was calculated as:

$\frac{\text{average deaths during 2020-2023}-\text{average deaths during 2018-2019}}{\text{average deaths during 2018-2019}}\times 100$

Crude mortality rates were used to reflect the overall population burden. Age adjustment was not performed because the primary objective was to describe observed temporal changes in raw mortality.

Analyses were descriptive in nature, focusing on temporal trends in annual mortality counts and crude mortality rates. Given the population-based design including all US deaths meeting the case definition, inferential statistical testing and sample-based hypothesis testing were not performed.

Ethical considerations

All data sources and classification systems used in this study are publicly available and free to access. The CDC WONDER database is an open-access governmental resource, and ICD-10 codes are part of the public domain classification system; therefore, no licenses or permissions were required. Because the dataset is de-identified and publicly available, institutional review board approval was not required.

## Results

From 2018 to 2023, a total of 32,738 deaths in the United States involved splanchnic thrombosis as an underlying or contributing cause of death.

Annual deaths increased from 4,819 in 2018 to 5,986 in 2023, representing the highest value during the study period.

**Figure 1:**
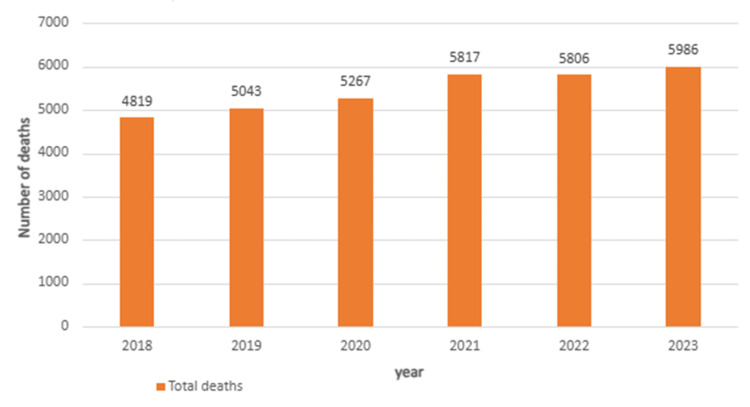
Splanchnic thrombosis mortality trends in the United States (2018-2023) This figure shows the total annual deaths observed during the study period. Bars represent absolute mortality counts per year, highlighting overall temporal patterns independent of population adjustment.

The pre-pandemic baseline average (2018-2019) was 4,931 deaths per year, compared with 5,719 deaths annually during 2020-2023, corresponding to a 16.0% relative increase. The largest year-to-year increase occurred between 2019 and 2021 (+15.3%) (Figure 1).

**Figure 2:**
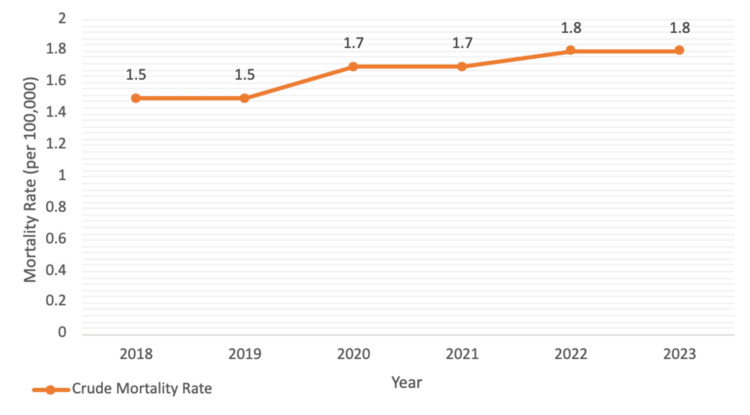
Crude mortality rate trend of splanchnic thrombosis (2018-2023) This figure shows the temporal trend in crude mortality rate per 100,000 population over the study period. The line demonstrates year-to-year changes in population-adjusted mortality without age standardization.

Crude mortality rates increased from approximately 1.5 deaths per 100,000 persons during 2018-2019 to 1.7-1.8 per 100,000 between 2021 and 2023 (Figure 2). Increases were observed in both sexes. Males accounted for approximately 47%-49% of deaths annually (Table 1).

**Table 1:** Annual mortality by sex (2018-2023) This table shows the annual number of deaths stratified by sex across the study period. Numbers represent total deaths for males and females each year, illustrating sex-based differences in mortality burden over time.

Year	Total deaths	Male deaths (%)	Female deaths (%)
2018	4,819	2,156 (45%)	2,663 (55%)
2019	5,043	2,322 (46%)	2,721 (54%)
2020	5,267	2,480 (47%)	2,787 (53%)
2021	5,817	2,822 (49%)	2,995 (51%)
2022	5,806	2,774 (48%)	3,032 (52%)
2023	5,986	2,820 (47%)	3,166 (53%)

## Discussion

The present national analysis identified a 16.0% relative increase in US deaths involving splanchnic thrombosis during the pandemic/post-pandemic period (2020-2023) compared with the pre-pandemic baseline (2018-2019). This finding indicates a sustained temporal rise in mortality during the COVID-19 era at the population level.

This analysis reflects population-level temporal changes observed during the pandemic period. The study design evaluated national mortality trends across defined time periods and did not include individual-level clinical data, infection status, or risk factor information. Accordingly, the analysis reflects changes in mortality patterns over time rather than the direct effect of severe acute respiratory syndrome coronavirus 2 (SARS-CoV-2) infection on clinical outcomes.

Several system-level factors may have contributed to the observed differences. Changes in healthcare utilization during the pandemic, including delayed presentation to medical care, reduced access to diagnostic imaging, and strained healthcare resources, were widely reported across acute medical conditions and may have resulted in more advanced disease at presentation. However, these potential contributors could not be directly evaluated within the current dataset.

Sex-stratified analyses demonstrated similar proportional trends among males and females, suggesting that the observed increase reflects broad population-level changes rather than sex-specific susceptibility.

The persistence of elevated mortality through 2023 suggests that the impact of the pandemic period may have extended beyond the initial surge years. Ongoing healthcare system recovery, delayed disease detection, or changes in clinical practice patterns may have contributed to this sustained trend. Continued surveillance is warranted to determine whether mortality rates return to pre-pandemic levels over time.

Prior clinical studies have reported increased rates of venous and arterial thrombotic events among hospitalized patients with COVID-19 [7-9]. In addition, experimental and pathological investigations have described endothelial injury, inflammatory activation, and dysregulated coagulation as potential contributors to COVID-associated coagulopathy [10-13]. Large observational studies and meta-analyses have further confirmed a high overall burden of thrombotic complications in this population [14-16]. However, the present analysis was not designed to evaluate biological mechanisms or infection-specific effects, and these observations should be interpreted only as contextual background rather than evidence of a causal relationship.

This study has several strengths. To our knowledge, it represents the first nationwide analysis examining temporal mortality trends involving splanchnic thrombosis across both pre-pandemic and pandemic periods. The use of a comprehensive national death registry provides broad population coverage and minimizes regional or institutional bias.

Several limitations should be acknowledged. Death certificate data may be subject to misclassification or underreporting, and the database does not include individual clinical variables, laboratory data, imaging findings, or treatment information. Importantly, causality between the pandemic period and changes in mortality cannot be established. Additionally, the analysis categorized time periods by calendar year; therefore, early 2020 may include both pre-pandemic and pandemic phases. Despite these limitations, national mortality databases remain valuable for identifying population-level trends and generating hypotheses for future research.

Overall, the findings demonstrate a measurable increase in deaths involving splanchnic thrombosis during the COVID-19 era. These results highlight the importance of continued monitoring of vascular outcomes and further investigation into factors contributing to temporal changes in mortality.

## Conclusions

Mortality involving splanchnic thrombosis increased in the United States during the COVID-19 era compared with the pre-pandemic years. This population-based analysis demonstrates a sustained temporal rise in deaths across the pandemic and post-pandemic period. Given the descriptive nature of the study, causal relationships cannot be established. These findings highlight the need for continued surveillance and further research to identify factors contributing to observed changes in mortality trends.
